# Phosphate level changes in oral cancer patients – recognizing the risk for refeeding syndrome

**DOI:** 10.1007/s00405-024-08972-8

**Published:** 2024-09-21

**Authors:** Suvi Silén, Erika Wilkman, Emilia Haukilehto, Arvi Keinänen, Antti Mäkitie, Johanna Snäll

**Affiliations:** 1 Department of Otorhinolaryngology, Head and Neck Centre, University of Helsinki and Helsinki University Hospital, Helsinki, Finland; 2 Department of Anaesthesiology and Intensive Care Medicine, University of Helsinki and Helsinki University Hospital, Helsinki, Finland; 3 Department of Oral and Maxillofacial Diseases, Clinicum, Faculty of Medicine, University of Helsinki, Helsinki University Hospital, Helsinki, Finland; 4 Research Programme in Systems Oncology, Faculty of Medicine, University of Helsinki, Helsinki, Finland

**Keywords:** Oral cancer, Oral squamous cell carcinoma, Malnutrition, Refeeding syndrome

## Abstract

**Purpose:**

Patients with oral squamous cell carcinoma (OSCC) often have difficulties in obtaining sufficient nutrition and may develop refeeding syndrome (RFS) during hospitalization. RFS may be fatal if not treated properly. This study clarified changes in perioperative phosphate levels and occurrence of RFS symptoms in OSCC patients to identify clinically notable predisposing factors for RFS in this specific patient population.

**Methods:**

A retrospective analysis included primary OSCC patients with microvascular free flap reconstruction. Patients with treatment for additional malignancy, hypoparathyroidism, and missing values of preoperative and/or postoperative plasma phosphate (P-Pi) concentration were excluded. The outcome variable was severe postoperative hypophosphataemia (mmol/l) during the postoperative period (P-Pi < 0.50 mmol/l). Predictor variables were age, sex, smoking, heavy alcohol use, diabetes, body mass index (BMI), weight, height, tumour site, tumour size, tracheostomy, nutritional route, and preoperative P-Pi concentration.

**Results:**

Of the 189 patients with primary OSCC, 21 (11%) developed severe hypophosphataemia. Of these patients, 17 (81%) developed RFS symptoms. Higher age (*p* = 0.01), lower patient height (*p* = 0.05), and no current smoking (*p* = 0.04) were significantly associated with postoperative hypophosphataemia. In multivariable regression analyses, higher age (OR 1.06 per year) and age over 70 years (OR 3.77) were independently associated with development of severe hypophosphataemia.

**Conclusion:**

Restoration of nutritional balance and close follow-up of electrolyte balance in the perioperative phase are necessary to prevent RFS, especially in patients with oral cancer requiring extensive reconstructions. Special attention should be focused on elderly patients since they are prone to this unnoticeable but potentially life-threatening electrolyte disturbance.

## Introduction

Head and neck cancers are a group of mostly squamous cell carcinomas, present in the upper aerodigestive tract and often affecting eating, and thus, the nutritional state of the patient [1]. Among cancers of the oral cavity, this is particularly common. Patients with oral squamous cell carcinoma (OSCC) are most often treated by surgery, followed by postoperative oncological treatment, if needed. As the tumours are resected with healthy tissue margins and the resection is often extended to the structures of the tongue and floor of the mouth, the process of eating and swallowing is likely to be considerably altered, at least transiently [2–4].

Major surgery of the oral cavity is highly radical and involves postoperative changes in diet, a prolonged swallowing rehabilitation, and consequently, the potential risk of malnutrition. Although it has been demonstrated that both compartmental surgery and extended hemiglossectomies involving the contralateral hemitongue can ensure satisfactory swallowing functional outcomes by preserving the minimal functional unit HSU (hyoglossus-styloglossus unit), the typical patient undergoing this surgery is often elderly, and postoperative rehabilitation may require several weeks for the adequate resumption of complete oral intake [3–5]. When the extent of the primary resection of the tumour calls for reconstructive surgery, the risks for malnutrition may be even higher. Enteral nutrition is often needed postoperatively [6]. Alcohol consumption is known to be strongly associated with both risk for OSCC and poor nutritional state [7, 8]. In addition, patients’ comorbidities are associated with preoperative protein metabolism [9, 10].

Refeeding syndrome (RFS), defined as a severe condition that affects patients with a previous period of malnutrition, followed by rapid feeding, induces hypophosphataemia and other electrolyte imbalances, leading to metabolic and clinical complications and eventual death, if left untreated [11]. Both enteral and parenteral refeeding can lead to RFS, but its prevalence seems to be higher in adults receiving enteral feeding [12]. Reported incidences in the literature vary markedly, depending strongly on the study population [13, 14]. No universally accepted definition of RFS exists, hindering assessment of its frequency.

Malnutrition leads to a low blood glucose level, which decreases insulin secretion, as glucagon secretion increases. This stimulates gluconeogenesis, leading initially to use of glycogen reserves, and later, to use of protein and fat [11]. If the condition continues, the system aims to save protein and decrease the use of ketone bodies, instead utilizing fatty acids. This results in severe intracellular mineral depletion, including phosphate, although in serum the mineral levels might still be normal [11]. When a starved body at refeeding receives high amounts of glucose, the body starts to synthesize glycogen, fat, and proteins again, requiring large amounts of electrolytes as well as their co-factors, e.g. thiamine. This leads to decreased phosphate levels in plasma, a phenomenon described as the “hallmark clinical sign” of RFS [11, 15]. In addition, magnesium and potassium are actively taken into the cells, leading to their sudden decrease in plasma and an excessive amount of fluid in the circulation [12]. This leads to metabolic complications and severe clinical manifestations, the first ones being peripheral oedema, respiratory insufficiency, and heart failure, followed by multiorgan failure and death [16, 17]. According to the 2020 criteria of the American Society of Parenteral and Enteral Nutrition (ASPEN), RFS develops during the first five days after initiation of refeeding [18–20]. Preoperative assessment of nutritional status of the patient and its optimization together with careful correction of electrolyte imbalances if present, should be done before surgery to avoid later development of RFS.

The purpose of this study was to clarify changes in perioperative phosphate levels in OSCC patients treated with resection of the tumour with microvascular free flap reconstruction and to evaluate their symptoms, potentially related to hypophosphataemia, during refeeding. We hypothesized that clinically notable predisposing factors can be found to predict and prevent postoperative RFS in this specific patient population.

## Materials and methods

### Patient material

We evaluated data of patients undergoing surgery for primary OSCC between January 2016 and December 2020 at the Head and Neck Centre, Helsinki University Hospital, Helsinki, Finland. Patients were retrieved from the multidisciplinary head and neck tumour board register, which covers treatment decisions for all OSCC patients.

### Inclusion and exclusion criteria

Only patients who needed microvascular free flap reconstruction of the resection site were included; patients with treatment for additional malignancy, hypoparathyroidism, and missing values of preoperative and/or postoperative plasma phosphate (P-Pi) concentration were excluded.

### Study design

The primary endpoint was severe postoperative hypophosphataemia (mmol/l) during the postoperative period (P-Pi < 0.50 mmol/l). P-Pi was measured daily up to the 5th postoperative day.

Predictor variables were age, sex, smoking, heavy alcohol use, diabetes, body mass index (BMI), weight, height, tumour site, tumour size according to TNM Staging of Lip and Oral Cavity cancers – AJCC 7th edition [21] and 8th edition [22, 23] valid at the time of diagnosis, tracheostomy received during the perioperative phase, nutritional route defined as early peroral (i.e. peroral nutrition started on 1st or 2nd postoperative day) or tube feeding (nasogastric tube or gastrostomy tube), and preoperative P-Pi concentration. The definition of heavy alcohol use was based on medical history and the background questionnaire and was deemed high if reported consumption was ≥ 23 doses (i.e. ≥287.5 g of alcohol) per week for men and ≥ 12 doses (i.e. ≥150 g of alcohol) per week for women.

Changes in P-Pi concentrations (mmol/l) during the perioperative phase were collected from the laboratory data. Symptoms and additional electrolyte imbalances of RFS patients were reported. In addition, the relationship between severe postoperative hypophosphataemia and 90-day mortality was analysed.

### Perioperative phosphate measurements and nutrition

Preoperative P-Pi (mmol/l) was examined in all patients 1–7 days before surgery, and postoperative P-Pi concentration was measured daily after surgery during the hospital period of up to five days, or occasionally even longer.

All patients underwent nutritional assessment before surgery, which included height and weight measurements and preoperative laboratory tests. Those who were malnourished received supplementary nutritional preparations before the surgical intervention according to the dietitian’s instructions. If a patient was thought to be at risk for RFS, nutritional supplementation was given cautiously.

### Statistical analyses

In statistical analyses, absolute numbers and proportions (%) or medians with interquartile ranges (IQR) of variables are presented. For continuous data, we used the Mann-Whitney U test for comparison of groups. For categorical data, we used Fisher’s exact test. We analysed the association of explanatory variables with the primary endpoint, hypophosphataemia below 0.5 mmol/l, in univariable analysis. We then included the prognostic factors with *p* ≤ 0.2 into forward conditional regression analyses to test independent associations with the primary endpoint. The following variables were entered into the forward conditional multivariable regression analyses: model 1: age, height, weight, current smoking, and tumour size (T1-T4); model 2: age over 70 years, height, weight, current smoking, and tumour size (T1-T4). We also analysed the association of hypophosphataemia with 90-day mortality using Fisher’s exact test.

We performed all statistical analyses with IBM SPSS Statistics 28.0 and 29.0 (IBM, Armonk, NY, USA) software.

## Results

**Fig. 1:**
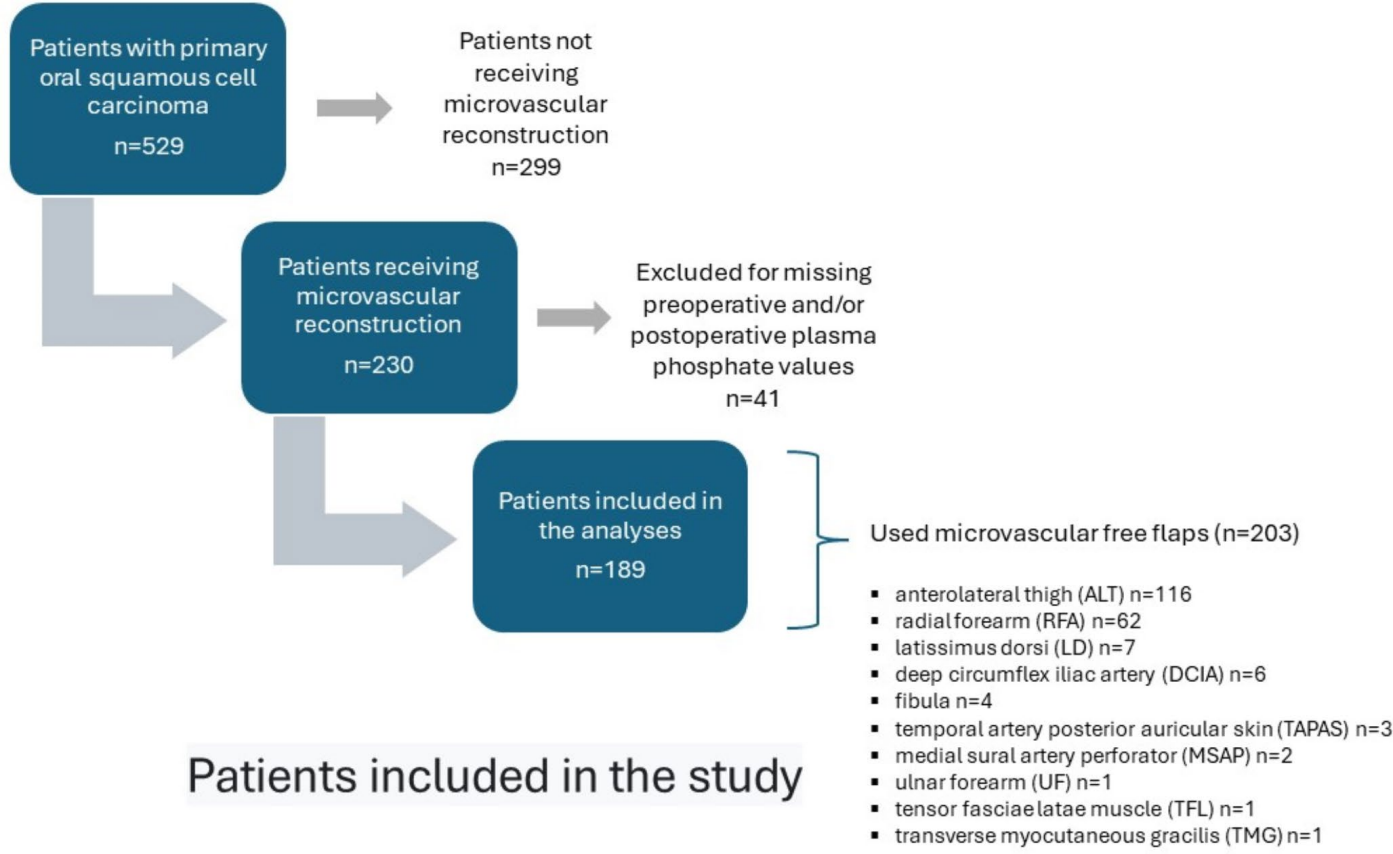
Patients included in the study

**Fig. 2:**
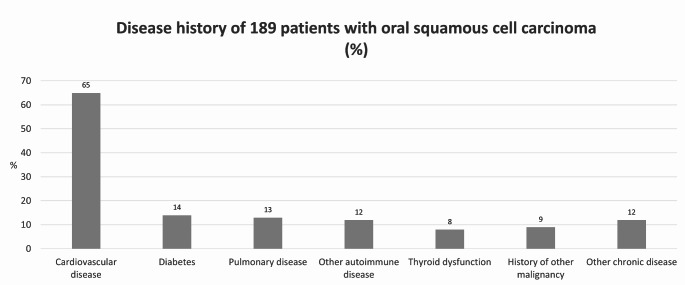
Disease history of 189 patients with squamous cell carcinoma (%)

Of the 529 patients with OSCC, 230 needed microvascular free flap reconstruction after the primary resection of the tumour (Fig. 1) All patients were preoperatively evaluated with computed tomography (CT) imaging and/or magnetic resonance imaging (MRI), their tumours were resected with surgical margins, and resections were complemented with microvascular free flap reconstruction. Ten different reconstructive flaps were used, the anterolateral thigh flap being the most common (Fig. 1). Forty-one patients were excluded due to missing preoperative and/or postoperative P-Pi values. None of the remaining patients had hypoparathyroidism or previous/additional malignancy. The most common comorbidities are shown in Fig. 2. Thus, 189 patients were included in the final analyses.

Age of patients ranged from 24 to 96 years (median 67 years, interquartile range (IQR) 67 years (60–75)), and 59% (112/189) were male (Table 1). Forty-eight patients (28%) reported heavy alcohol consumption. Early peroral nutrition after surgery was rare (7/189, 4%), i.e. the great majority received tube feeding postoperatively (96%). Patients’ median BMI (24.5) indicated normal weight in general, however, BMI varied from 15.5 to 47.8.

**Table 1:** Descriptive statistics of 189 patients undergoing microvascular free flap reconstruction for oral squamous cell carcinoma

	*n*	% of all
**All**	189	
**Age (years)**		
Median	67.0	
IQR	59.5–75.0	
**Sex**		
Male	112	59.3
Female	77	40.7
**Smoking**		
Yes	98	51.9
No	91	48.1
**Diabetes**		
Yes	27	14.3
No	162	85.7
**Tumour site**		
Tongue	66	34.9
Gingiva	57	30.2
Floor of mouth	32	16.9
Buccal mucosa	24	12.7
Palate	10	5.3
**T-class**		
1	25	13.2
2	53	28.0
3	45	23.8
4	66	34.9
**Tracheostomy**		
Yes	99	52.4
No	90	47.6
**Postoperative nutritional route**		
Tube feeding	182	96.3
Early peroral	7	3.7
**Preoperative phosphate (mmol/l)**		
Median	0.97	
IQR	0.87–1.08	
Body mass index *		
Median	24.5	
IQR	21.7–27.3	
Body mass index: weight (kg) / [height (m)]^2^		
IQR = Interquartile range		

**Fig. 3:**
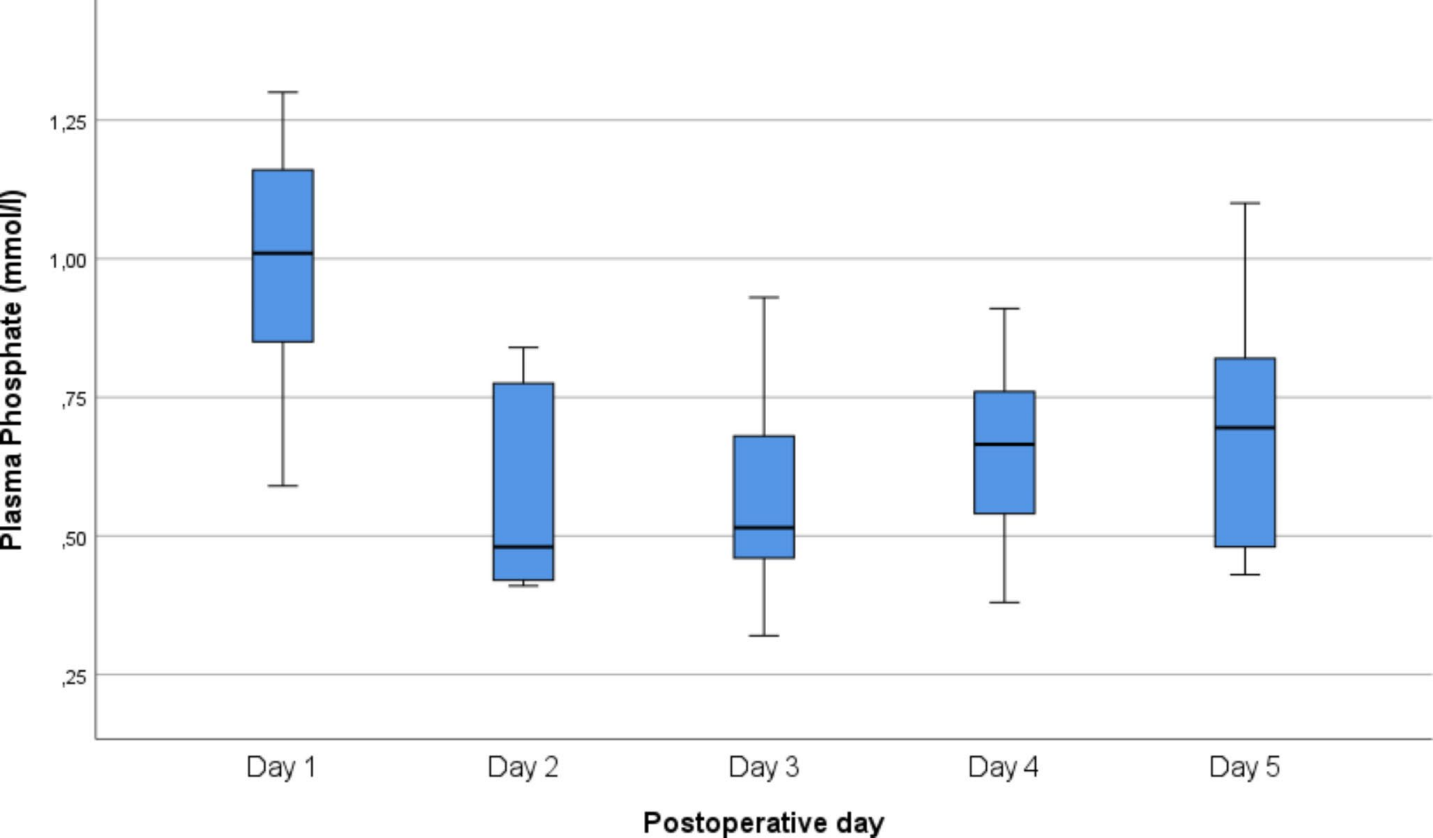
Changes in plasma phosphate levels during the first postoperative days in oral squamous cell carcinoma patients

The decrease in P-Pi (mmol/l) occurred during postoperative days 2–5 and in two patients on postoperative days 6 and 7. P-Pi concentration of these severe hypophosphataemia patients ranged from 0.33 mmol/l to 0.49 mmol/l (median 0.44 mmol/l). The phosphate level decrease was greatest on the 2nd and 3rd postoperative days (Fig. 3).

In all, 21 patients (11%) developed severe hypophosphataemia postoperatively (Table 2). In univariable analyses, higher age (*p* = 0.01), lower height (*p* = 0.05), and no current smoking (*p* = 0.04) were significantly associated with development of postoperative hypophosphataemia. Postoperative 90-day mortality did not differ significantly in patients with and without hypophosphataemia (19% vs. 7.1%, *p* = 0.08).

**Table 2:** Association between predictor variables and 90-day mortality and severe postoperative hypophosphataemia in oral squamous cell carcinoma patients undergoing microvascular free flap reconstruction

	Patients with severe hypophosphataemia			Patients without severe hypophosphataemia		*p*-value
	*n*	%		*n*	%	
	21	11.1		168	88.9	
**Age (years)**						
Median	73.0			66.0		0.01
IQR	68.0–81.0			62.0–74.8		
	**n**	**% of n**		**n**	**% of n**	
**Sex**						0.83
Male	12	10.7		100	89.3	
Female	9	11.7		68	88.3	
**Current smoking**						0.04
Yes	6	6.1		92	93.9	
No	15	16.5		76	83.5	
**Heavy alcohol use**						0.60
Yes	4	8.3		44	91.7	
No	17	12.1		124	87.9	
**Diabetes**						0.76
Yes	3	11.1		24	88.9	
No	18	11.1		144	88.9	
**Tumour site**						0.34
Tongue	10	15.2		56	84.8	
Gingiva	7	12.2		50	87.7	
Floor of mouth	1	3.1		31	96.9	
Buccal mucosa	3	12.5		21	87.5	
Palate	0	0.0		10	100.0	
**T-class**						0.14
1	1	4.0		24	96.0	
2	3	5.7		50	94.3	
3	9	20.0		36	80.0	
4	8	12.1		58	87.9	
**Tracheostomy**						0.83
Yes	11	11.1		88	88.9	
No	10	11.1		80	88.9	
**Postoperative nutritional route**						0.57
Tube feeding	20	11.0		162	89.0	
Early oral	1	14.3		6	85.7	
**Preoperative phosphate (mmol/l)**						0.23
Median	0.95			0.98		
IQR	0.75–1.07			0.89–1.08		
Body mass index *						0.68
Median	24.2			24.6		
IQR	21.9–26.5			21.7–27.5		
**Weight (kg)**						0.20
Median	70.0			72.0		
IQR	59.0–75.0			62.0–81.8		
**Height (cm)**						0.05
Median	167.0			172.0		
IQR	158.0–173.0			165.0–178.0		
**90-day postoperative mortality**						0.08
Yes	4	25.0		12	75.0	
No	17	9.8		156	90.2	
*Body mass index: weight (kg) / [height (m)]^2^						
IQR = Interquartile range						

**Fig. 4:**
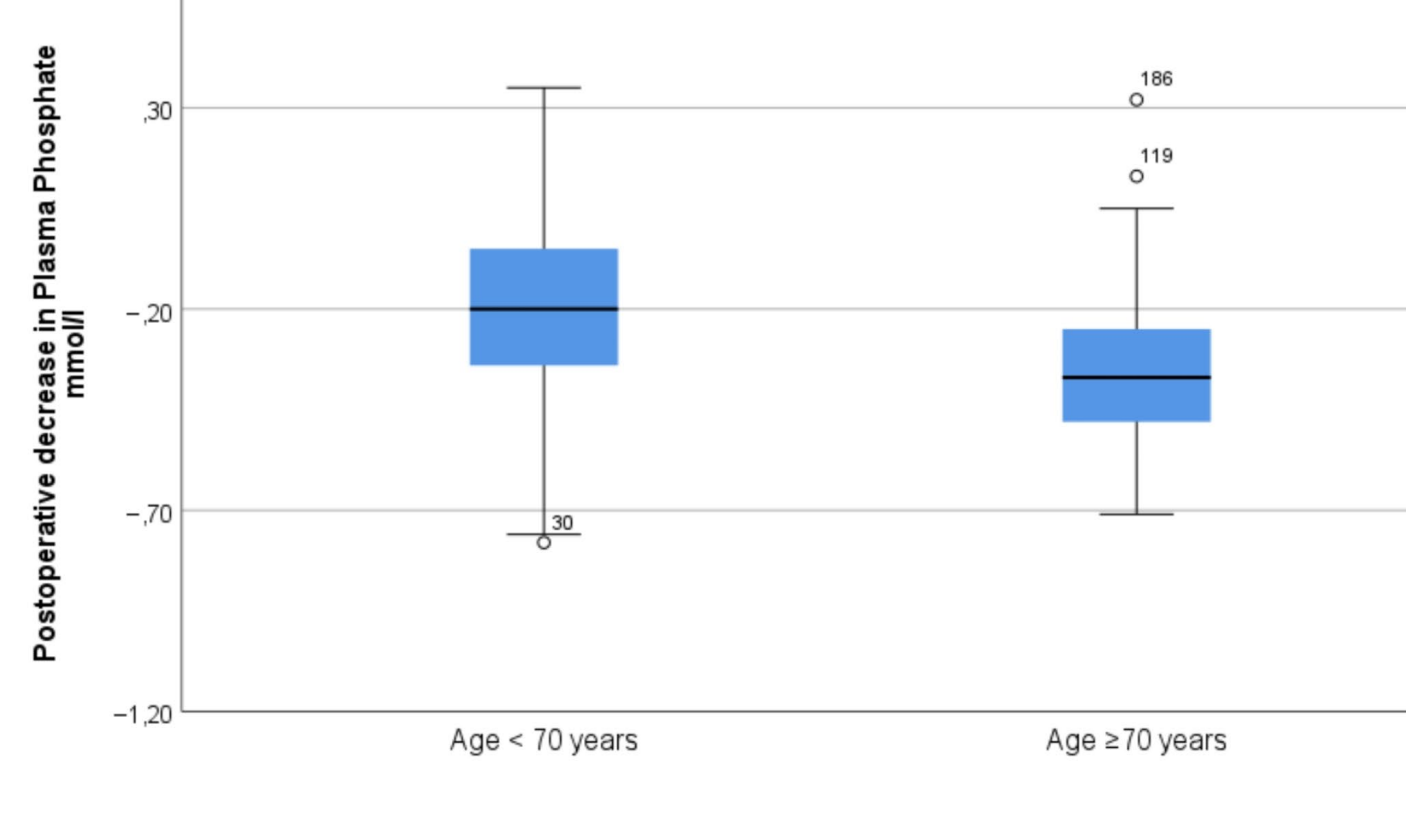
Postoperative decrease in plasma phosphate levels according to age group in oral squamous cell carcinoma patients

In the forward conditional multivariable regression analyses, higher age (OR 1.06, 95% CI 1.02–1.11) per year or alternatively age over 70 years (OR 3.77, 95% CI 1.39–10.20) was independently associated with development of hypophosphataemia (Fig. 4).

Of the 21 patients with severe postoperative hypophosphataemia, 7 (33%) had low albumin levels in plasma preoperatively. Seventeen with low P-Pi (81%) had symptoms related to RFS, including extreme tiredness, nausea, vomiting, dyspnoea, delirium, bradycardia, and other arrhythmias (Table 3). One patient suffered from cardiac arrest and received successful cardiopulmonary resuscitation. Seven patients (33%) had hypokalaemia (< 0.34 mmol/l) and 9 patients (43%) hypomagnesaemia postoperatively. Fourteen (67%) of the 21 patients received either peroral or intravenous phosphate.

**Table 3:** Findings in 21 oral squamous cell carcinoma patients with postoperative hypophosphataemia

	*n*	% of 21	
**Symptom**			
fatigue	10	48	
disorientation	6	29	
nausea and/or vomiting	4	19	
diarrhoea	1	5	
headache	1	5	
stomach ache	1	5	
dizziness	1	5	
cardiac arrhythmia	7	33	
atrial fibrillation	2	10	
bradycardia	1	5	
bradycardia and atrioventricular block	1	5	
asystole **	1	5	
QT-time prolongation	1	5	
impairment of lung function and heart failure ***	1	5	
symptomless	4	19	
**Electrolyte changes and renal function**			
decreased GFR	3	14	
hypokalaemia	7	33	
hypernatraemia	3	14	
hypomagnesaemia	9	43	
hypocalcaemia	9	43	
**Preoperative nutritional stage**			
hypoalbuminaemia	7	33	
*requiring medical treatment			
**led to successful resuscitation			
***led to death			
GFR = glomerular filtration rate ml/min/1.73m^2^			

## Discussion

In this study, we clarify changes in perioperative phosphate levels and occurrence of RFS in an OSCC cohort treated with primary resection of the tumour and reconstructive surgery with microvascular free flap.

The risk of developing RFS is obvious in OSCC patients for several reasons. In addition to the malignancy itself, tumours situated in the oral region cause challenges in nutrition. Alcohol abuse is common in OSCC patients, which may affect the patient’s nutritional status. Reviewing RFS in OSCC patients and comparing results with previous studies are complicated by the lack of both a standardized definition for the syndrome and treatment recommendations.

In the present study, the resections of different surgical sites aimed for a healthy tissue margin, and all patients received a free flap reconstruction. Our study shows that perioperative phosphate level evaluation should be routine in major OSCC surgery. Low phosphate levels (< 0.5 mmol/l) were found postoperatively in 11% of patients, 81% of whom had symptoms possibly linked to refeeding (i.e. 9% of all OSCC patients were symptomatic). Our hypothesis was confirmed since a clinically notable predictor was found. Surprisingly, previous heavy alcohol use was not related to hypophosphataemia, nor did BMI explain phosphate level changes. Instead, hypophosphataemia was significantly more common in elderly patients.

The phenomenon of ageing as a risk factor for RFS has been observed previously, although not in head and neck cancer patients. Among other things, studies performed in internal medicine and in hospitalized corona patients have shown a trend towards a higher risk of RFS in the elderly [13, 24, 25]. According to our results, the ratio of OSCC patients developing hypophosphataemia was markedly different in patients aged under and over 65 years. When a cut-off of 70 years was used, postoperative hypophosphataemia was found in 18% of elderly patients. The occurrence was threefold higher relative to younger patients.

The occurrence of OSCC is accentuated in older age groups [26], and postoperative complications related to OSCC surgery are associated with underlying diseases in elderly patients [27]. Elderly patients have diminished tolerance for stress factors and are likely to have comorbidities associated with RFS risk [13, 28]. Further, RFS can delay and complicate recovery, especially in the elderly and in patients with comorbidities [28]. Nevertheless, RFS in the older population may go unrecognized and undiagnosed because the symptoms are non-specific and resemble those of frailty in the elderly (e.g. weakness, confusion, poor mobility). Special attention must therefore be paid to nutrition and RFS in older OSCC patients, especially during immediate postoperative recovery.

Preoperative assessment of nutritional status is essential in OSCC patients, however, evaluation of RFS risk can be difficult. In this study, preoperative hypoalbuminaemia was a weak indicator of RFS. It was found in only one-third of hypophosphataemia patients. Low BMI was not associated with RFS. However, previous changes in weight should be considered when assessing RFS risk. Unintentional weight loss of > 15% in the past 3–6 months is a risk factor for developing refeeding problems [11]. Thus, in addition to preoperative hypoalbuminaemia evaluation, a meticulous medical history is essential in assessment of OSCC patients’ nutritional status to identify those patients whose nutritional status should be corrected before surgery. The nutritional assessment is essential in preventing complications and mortality. Although lacking statistical significance, 90-day mortality was notably higher in patients with hypophosphataemia than in those without hypophosphataemia (19% vs. 7%).

Based on our research, it cannot be confirmed that all listed symptoms (Table 3) were specifically related to phosphate balance, as these symptoms are common in the postoperative phase in intensive care patients. On the other hand, changes in other electrolyte levels with hypophosphataemia were found in only some patients. Seven (33%) of 21 patients also had hypokalaemia. One of these patients had asystole, and the patient was resuscitated. Nine patients (43%) had hypomagnesaemia, and the same number of patients had hypocalcaemia. Hypocalcaemia is known to induce secretion of parathyroid hormone (PTH) in the parathyroid glands, which affects the renal handling of phosphate, further decreasing the levels of phosphate in serum [29]. Low levels of vitamin D, previously shown to be present in head and neck cancer patients, additionally decrease the absorption of calcium in the intestine, leading to hypocalcaemia [30]. Therefore, vitamin D and calcium substitution, if needed, lowers the risk of hypophosphataemia and RFS. Measuring pre- and post-treatment PTH in these patients could be considered, also taking into account that these patients often have large neck metastases that may possibly impact blood flow in the parathyroid gland area, potentially affecting PTH levels. Only one patient (5%) had depletion of all phosphate, potassium, and magnesium. Unlike the depletion of phosphate and potassium, the cause of hypomagnesaemia in the refeeding situation is not entirely clear, and many cases of hypomagnesaemia are asymptomatic; however, when severe, hypomagnesaemia has been associated with *torsades de pointes* [31]. Overall, general electrolyte balance must be carefully evaluated in OSCC patients in the postoperative period. Hypophosphataemia occurs with various electrolyte disturbances but also as a separate issue. Thus, our results strengthen the idea of hypophosphataemia being the most important electrolyte disturbance in RFS.

Due to the retrospective design of our study, mild hypophosphataemia-related symptoms and findings may have gone unreported, leading to an underestimation of symptoms. An additional limitation of the study is that preoperative nutritional status was not systematically recorded. Careful and systematic preoperative assessment of nutritional status includes dietitian assessment, measurements of electrolyte balance and prealbumin level in addition to height and weight and history of previous weight loss. We retrospectively observed that some of the data were incompletely recorded, which must be listed as limitations of the study. Regarding alcohol use, the reported doses are probably also lower than the actual ones since patients tend to underestimate their alcohol consumption and clinicians do not necessarily remember to ask or record these details [32]. In future, prospective studies of nutritional status and RFS in the OSCC patient population would provide valuable information for clinical work and improve patient perioperative recovery.

Phosphate levels decreased imperceptibly in OSCC patients. The most notable predictive factor was age, as older patients were prone to this otherwise unpredictable electrolyte disturbance. Symptoms related to hypophosphataemia, often abnormal fatigue, nausea, and delirium, were common. These findings emphasize the importance of raising awareness of this issue among health care professionals participating in the care of OSCC patients.
